# Youth perspectives: experiences with a multidisciplinary weight management service, a qualitative study

**DOI:** 10.1186/s12887-025-06239-7

**Published:** 2025-11-18

**Authors:** Shen Meei Chia, Slavica Krstic, Fang Lin, Sarah Dennis, Faye Southcombe

**Affiliations:** 1 Primary and Community Health South Western Sydney Local Health District, Sydney, 5 Thomas Rose Drive, Rosemeadow NSW 2560 Australia; 2 The University of Sydney, Sydney, Australia; 3 Ingham Institute of Applied Medical Research, Sydney, Australia

**Keywords:** Adolescent, Youth, Perspective, Experience, Obesity, Paediatric, Weight management, Service delivery, Multidisciplinary

## Abstract

**Background:**

This study aims to explore adolescents’ perspectives on their experiences with Growing Healthy Kids (GHK), a multidisciplinary weight management service, evaluating their overall satisfaction and identifying areas for improvement in service delivery, acceptability, and effectiveness.

**Methods:**

A qualitative study using semi-structured interviews explored the perspectives of adolescents living with obesity who had received weight management services through the GHK Service. Interviews were conducted between July 2022 and July 2023 by a non-treating clinician, either face-to-face or by telephone, with written informed consent obtained prior. Interviews were digitally audio-recorded and transcribed verbatim. Thematic analysis was completed in NVivo by three independent researchers. Ethics approval was granted by the South Western Sydney Local Health District (SWSLHD) Human Research and Ethics Committee.

**Results:**

A total of 22 adolescents were interviewed. Thematic analysis of the interviews revealed four key themes: (i) initial experiences and feelings, (ii) perceptions of service delivery, (iii) impact of the service, and (iv) recommendations for improvement.

**Conclusions:**

This study emphasises the benefits of a multidisciplinary tailored interventions which meet the unique needs of adolescents in weight management services. It identifies emotional challenges such as anxiety and judgment, highlighting the importance of clear communication, empathetic care, and supportive environments. Participants expressed a need for long-term support, flexible appointments, and access to resources like gym equipment. These findings suggest that extending access to weight management services and offering adaptable care can enhance the effectiveness of programs like GHK, promoting sustained engagement and positive health outcomes for adolescents.

**Supplementary Information:**

The online version contains supplementary material available at 10.1186/s12887-025-06239-7.

##  Background

In Australia, over a quarter (27.7%) of children aged 5 to 17 years were classified as overweight or obese in 2022 [1]. The prevalence is even higher among priority population subgroups [2–4]. South Western Sydney Local Health District (SWSLHD) has one of the largest urban Aboriginal populations, as well as Culturally and Linguistically Diverse (CALD) groups, and socioeconomically disadvantaged populations [5, 6]. This health district has the highest rates of childhood overweight and obesity in New South Wales (NSW), at 37.3% [6].

Childhood obesity increases the immediate and lifelong risk of developing chronic diseases such as diabetes, heart disease, obstructive sleep apnoea and fatty liver disease [7]. It also negatively impacts psychosocial health, including low self-esteem, body dissatisfaction [8] and an increased risk of experiencing bullying [9]. A cross-sectional study of the children receiving obesity treatment through the Growing Healthy Kids (GHK) service in SWSLHD showed that 75% of children had prediabetes, 52% had an abnormal lipid profile, and 30% had an elevated blood pressure [10].

Given that 80% of adolescents living with obesity will remain obese in adulthood without treatment, early and effective treatment is crucial [11]. Adolescents are a population with unique needs, and their experience and satisfaction with the GHK service are crucial to optimise treatment and clinical outcomes and minimise attrition. The GHK service provides a multidisciplinary and multicomponent lifestyle intervention including a range of dietary, exercise, and behavioural interventions. The GHK service has been evaluated and has demonstrated clinically important reductions in body mass index (BMI), improvement in metabolic health, child behaviour and overall wellbeing [12]. While there is a body of evidence supporting the effectiveness of a multidisciplinary service in treating obesity [13], there is limited research to explore the perceptions and experiences of participants in multidisciplinary weight management services [14]. This gap is particularly evident in the studies focusing on lower-income families and those from CALD backgrounds [15, 16]. By obtaining a more in-depth understanding from the participants through this qualitative study, we aim to determine if the model of service delivery meets the needs and expectations of its clients. The objective of this study was to investigate adolescents’ overall satisfaction of the service and identifying potential improvements in the acceptability and effectiveness of the service.

## Methods

This is a qualitative study that uses a descriptive qualitative design which aims to explore and understand participants’ experiences in their own words without imposing a theoretical framework Doyle, McCabe [17]. This approach is commonly used in health research to generate practical, experience-based insights that can inform service delivery. Semi-structured interviews were conducted with participants aged 12 to 17 years who are living with obesity and attending the GHK service.

GHK is a 24-month multidisciplinary publicly funded service, led by a paediatrician, clinical nurse specialist, social worker, clinical psychologist, exercise physiologist and dietitians. Individual sessions were provided to all participants of GHK. Participants were recommended to visit a treating clinician at a minimum monthly basis, however this is tailored according to individual needs and availability.

Between July 2022 and July 2023, adolescents who attended the service for longer than six months were invited to participate in the study. Eligible participants and their parents were approached by the research team directly after they visited the service or were contacted via telephone. The study was explained to them, and an invitation to participate was offered. Adolescents were excluded if they were non-verbal due to autism spectrum disorder or other developmental disorders.

All eligible participants were provided with a participant information sheet that outlined the purpose of the research. All participants provided their written informed consent to participate, and for those under the age of 16, their parents signed the consent form. Participants were not remunerated.

Interviews were conducted face-to-face at the facility they were attending or over the telephone. Participants were informed that the discussions would be audio-recorded, and their responses would be kept confidential and deidentified. Identifiable responses were only accessible to the research team. Most interviews were conducted with participants alone, but in some cases, a parent was present.

The interviews were conducted by one of three clinicians who had experience in communicating with children living with obesity. Before data collection, the interviewers practiced conducting semi-structured interviews with a focus on using open-ended questions, and encouraging participants to elaborate freely. The interview guide was pilot tested with clinicians, as well as two clients who were not participants of the study to ensure clarity, neutrality and appropriateness of follow up questions (refer to Appendix A for interview guide). To minimise potential biases, the interviews were conducted by clinicians who were not involved in the participant’s care. However, due to an oversight, one interview was conducted by the participant’s treating dietitian.

The research team included a clinical nurse specialist (SK), three dietitians (SC, FL, FS) who all work within the GHK service, and health services researcher (SD) who is external to the service. The clinical members of the team have extensive experience in managing paediatric obesity, which helped build rapport with participants and encouraged open dialogue during the interviews. Our clinical backgrounds and familiarity with the service may have influenced the interpretation of participants’ feedback, particularly with a focus on clinical aspects of their experience. We used a standard interview guide to ensure consistency and had regular team debriefs with a senior researcher (SD) to further support impartiality during data collection and analysis.

Each interview lasted approximately 10 to 25 min using an interview guide developed by the research team. The guide was tested prior to use to ensure that the questions were clear and appropriate. The testing phase also facilitated the development of probing questions that encouraged participants to provide more detailed responses. All interviews were conducted in English as all participants spoke fluent English.

After conducting seven interviews, the research team met to discuss the sampling and identified that the recruitment was biased towards males (six males and one female). To address this, we purposively sampled more female participants. The research team met regularly to debrief following the interviews, which allowed the team to determine when data sufficiency had been achieved. Data sufficiency was defined as the point at which no new information or insights were gained from the participants. As a result, recruitment stopped once data sufficiency was achieved, resulting in 22 interviews being conducted. The demographic data, including the participants’ age, sex, CALD and Aboriginal status, were obtained from electronic medical record (eMR) and self-reported information by the participants and their families at initial consultation. CALD status was defined as participants born in non-English speaking countries, and/or those who spoke a language other than English at home [18]. All interviews were digitally recorded and transcribed verbatim.

Two researchers (FL, SK) read all the transcripts carefully to obtain an overall understanding of the interviews. To develop a coding framework, we used an inductive approach and utilised the thematic analysis method proposed by Nowell et al. [19]. One researcher (SK) coded the transcripts line by line in NVivo (QSR International Pty Ltd. (2018)). NVivo was used as a data management tool to organise and facilitate coding. This coding process allowed codes and themes to be systematically identified and developed from the data. The research team met regularly to discuss the coding, review coded transcripts and resolve discrepancies. Subthemes were developed by grouping related codes into border categories based on team consensus. The coding and discussions were supervised by an experienced qualitative researcher (SD). This approach ensured that the analysis was closely tied to the participants’ perspective, ensuring the accuracy and reliability of the results.

Ethics approval was obtained from SWSLHD Human Research and Ethics Committee (2019/ETH12871).

## Results

Of the 30 adolescents approached, 22 consented to participate in the study and were interviewed. Reasons for declining to participate were not explored. The demographic characteristics of the adolescents are detailed in Table 1. The demographics of this sample reflect the population of children seen by the GHK service [10]. A larger proportion of males (68.2%, *n* = 15) were interviewed, likely reflecting the greater proportion of males enrolled in the GHK service compared to females. The average age of the participants was 14.9 years (SD = 1.6, range 12 to 17 years). The majority of participants were from a CALD background (59.1%, *n* = 13) and 9.1% (*n* = 2) identified as Aboriginal.

**Table 1:** Demographic data

Participant	Age (years)	Sex	Background
Participant 1	17	Male	Australian - Caucasian
Participant 2	14	Male	Australian - Caucasian
Participant 3	17	Male	Australian - CALD
Participant 4	15	Female	Australian - CALD
Participant 5	15	Female	Australian - CALD
Participant 6	16	Male	Australian - CALD
Participant 7	16	Male	Australian - Aboriginal
Participant 8	13	Male	Australian - CALD
Participant 9	16	Male	Australian - CALD
Participant 10	14	Male	Australian - CALD
Participant 11	17	Male	Australian - CALD
Participant 12	14	Male	Australian – Caucasian
Participant 13	15	Female	Australian - Aboriginal
Participant 14	12	Male	Australian – Caucasian
Participant 15	16	Male	Australian – Caucasian
Participant 16	13	Female	Australian - CALD
Participant 17	12	Male	Australian - CALD
Participant 18	15	Female	Australian - CALD
Participant 19	17	Male	Australian - CALD
Participant 20	14	Female	Australian - CALD
Participant 21	17	Female	Australian - Caucasian
Participant 22	12	Male	Australian - Caucasian

*CALD* Culturally and linguistically diverse

Thematic analysis of the interview transcripts identified four main themes: the client’s initial experiences and feelings, service delivery, impact and recommendations. These are described in detail below.

### Theme 1: client’s initial experiences and feelings

This theme was developed to include two subthemes: initial impressions and perceived need for the service.

### Subtheme 1: initial impression

Participants shared their emotions, thoughts, and expectations about engaging with the GHK service for the first time. These experiences were shaped by unfamiliarity, personal apprehension, and concerns about judgment.

Participants expressed a range of emotions before starting the service. Many participants reported feeling nervous before engaging with the service, primarily due to the novelty and uncertainty of potential outcomes.

“I felt a little nervous…. because it was a first-time experience” (P2).

“Definitely nervous and stressed… because it was something new” (P10).

“Nervous… something new never done” (P17).

“Because I didn’t know how my dietitian and all that were going to be …I didn’t know if they were going to be like strict, mean” (P22).

“It was just my own self-conscious mind. It’s always expecting the worst” (P2).

Some participants attributed nervousness to meeting new people:

“Normally I feel nervous with new people” (P17).

While some reported feeling uncertain about the potential changes, others were concerned about being judged based on their weight or appearance. Some were optimistic about the changes they might make.

“When I first started, I was a bit uncertain… Because I didn’t know if it was going to work or not. I’m not used to change.” (P15).

“I was worried about how much I weigh… I was worried I was going to be judged. I just didn’t feel well with my body” (P14).

“I felt excited like… to lose weight… To be fit and healthy” (P19).

“I wanted to learn, different ways that I could also speed that up. See how well I was eating and stuff like that” (P 1).

Clear communication and comprehensive explanations about the program were instrumental in alleviating participants’ anxiety. Some would have liked more explanation prior to their first appointment and others believed that their nervousness was inevitable regardless of the information provided.

“It was explained properly, there was a lot of information about the program on that day…. So when I was listening to all the information, it was kind of making me feel a lot better” (P5).

“I think having more details about the service beforehand might have helped” (P12).

“I’m not really sure, I felt like I’d be nervous either way” (P20).

Despite initial stress, many participants noted that their nervousness diminished as they engaged in the program and received support.

“No, I think it just took me a bit of getting used to, like just doing something new” (P15).

“It wasn’t really much of you guys. I think it was me” (P5).

“But when you get there, it’s different” (P2).

After engaging in the service, participants generally experienced a positive shift in their outlook.

“A lot of the doctors were very friendly. It wasn’t that nerve-wracking when we went in” (P18).

“It just took me like a bit of getting used to, like doing something new” (P16).

“When you get there, it’s different” (P2).

“At the beginning, not confident at all… But now that I’ve been doing it for nearly twelve months, it’s like really helped” (P12).

These insights emphasise the value of clear communication and providing adolescent targeted and focused information about the service in advance to help ease participants’ concerns and to reduce the risk of them not turning up at all.

### Subtheme 2. Perceived need for the service

This subtheme was identified to reflect participants’ perceptions of the service’s necessity, examining both personal and external motivations that influenced their willingness to engage. Many participants articulated a personal awareness of the need for the intervention to address their weight concerns.

“I was putting on weight. I needed to figure out how to lose some by exercise and stuff” (P14).

“I was not healthy back then, not in the slightest” (P2).

Participants said that their family members had varying viewpoints about the participants’ need for weight management service.

“Yes, my mum definitely thought so…. but I’m not sure about my dad” (P2).

“My parents obviously thought I should do something because my weight was increasing quite quickly” (P4).

My parents thought that maybe I did need it” (P6).

Some participants perceived the service as an inconvenience initially, however this view changed positively over time, and they became empowered to make change.

“To be honest, in the beginning, it felt like more of an inconvenience than anything. But as the years progressed, I found that I’m getting a lot better at understanding what to do and, you know, deciding for myself” (P11).

### Theme 2: service delivery

This theme delves into the nuances of service delivery, capturing two distinctive subthemes: mode of delivery and interaction, and client-centred care.

#### Subtheme 1: mode of delivery and interaction

Participants expressed diverse preferences for how services were delivered and session formats. These preferences often reflected their comfort levels, personalities and past experiences. Overall, there was a clear preference for individual sessions. They emphasised the benefits of individualised care, privacy and the ability to establish stronger connections with the healthcare professionals (HCPs).

“Individual sessions are better because sometimes it’s embarrassing to talk in front of a group” (P13).

“It’s just easier, it is better, it is a bit more straight forward” (P15).

“I feel like individual, you get to know the person more… So it feels better to just be one-on-one with the dietitian or whoever’s seeing me” (P10).

Participants highlighted how individual sessions made them feel more at ease and better understood. One-on-one interactions also provided a safe space to share personal concerns, which many felt would be difficult in a group setting.

“I feel like in a group setting, I wouldn’t really say much. I wouldn’t really be interested in that” (P20).

“It’s better because I’m afraid to ask questions in a group. Individual is just easier and more straightforward” (P15).

“I wouldn’t really be open to a group session. It’s intimidating” (P11).

“It would be a bit embarrassing” (P18).

Some participants explained that group settings could cause stress or anxiety due to social dynamics, fear of judgment or prior negative experiences, such as bullying.

“When it’s with a group it can be stressful dealing with all that people” (P 2).

“I’m not really the type of person who wants to do sports with other people” (P 16).

“Definite no for group session… not for me, not with weight. Social anxiety about my weight. I don’t think I’d be comfortable with a group of kids talking about my weight… I got sick because I was so severely bullied from other kids” (P21).

“Because the kids that are a part of the group, you know, they could talk behind your back about you” (P22).

“I don’t like being around people much” (P14).

While the prevailing sentiment favoured individual sessions, one participant presented an alternative perspective, finding comfort in group settings.

“A group session. Because I’m not really good at talking to someone one-on-one” (P8).

Some participants found it convenient and efficient to see multiple clinicians during a single visit, saving time and effort by addressing various needs all at once.

“It was good. It saved me from like having to come back. Everything was done at once which was good” (P4).

Participants also provided insights into their preferred modes of interaction. Face-to-face interactions were consistently favoured by most participants, who cited benefits such as better communication, more personalised guidance, and a stronger sense of connection and accountability.

“Generally better because they can see our progress; they can give us proper advice…. it feels more comfortable” (P3).

“When it’s face-to-face, they can see how you’re doing, and it feels more comfortable” (P20).

Telehealth was available in the GHK service, but many of those interviewed found that video calls were less engaging, with one participant observing their parent often took over during virtual sessions. However, they acknowledged the utility of video calls, especially when physical meetings were not possible.

“Um when we done the video calls it was mostly my mum just talking…., but I like more face to face” (P20).

“The zoom call probably ok because it’s like face-to-face. If unable to attend face to face” (P8).

“I did use telehealth sometimes and it was pretty good” (P7).

Some also highlighted the convenience of mixed approaches involving both face-to-face and video calls. This was seen as a practical solution, particularly for managing time and accommodating family or school commitments.

“I felt like it was actually less time-consuming” (P16).

“So that would make it easier for schooling-wise and for mum and dad as well” (P18).

The options for home visits were viewed with mixed responses. Some participants valued that HCPs could gain insight into their living conditions and home environment, but others were not so keen.

“I guess it would be kind of a good way to get the staff members to understand the environment where they live”(P11).

“Home visits are much better…. If you come to house it’s much easier” (P9).

#### Subtheme 2: Client-centered care

The participants consistently highlighted positive aspects of client-centred care, highlighting the supportive and respectful approach of HCPs. Staff were described as approachable, friendly and attentive, creating an environment where participants felt comfortable and valued. The approachability of HCPs made it easier for participants to share their concerns and feel listened to. Many felt genuinely cared for and supported throughout their journey.

”Everyone was really nice; they were really respectful; they were, like, super kind and like really straight forward with things” (P1).

“Yeah they were helpful… very respectful. helpful like when they talk” (P19).

“I felt comfortable….if I’m not comfortable around someone, it can be hard to concentrate … but I was really motivated to get to where I am today (P15).

“I found them very caring and they were like actually trying to help throughout my journey” (P9).

“Felt I was listened to”(P2).

HCPs were described as always maintaining a high level of professionalism and participants appreciated their careful and factual communication.

“100% always professional, they’re always kind with their words, they always watch out what they’re saying to make sure that it’s not hurtful or anything…., every word that they say is factual. Every word that they say is related to what I’m experiencing, it’s to help me” (P11).

They also highlighted how the information provided was easy to understand, ensuring that they felt informed about their care.

“The staff was really nice like the two Doctors that I had, they were really good with kids, they weren’t too straight forward they kind of dumbed it down a bit so you could understand. …they actually like explained… treated you really nicely” (P15).

“They were approachable; they were reasonable, the exercises and advice was something that I was able to do …I think it’s generally good” (P3).

Support from clinicians was consistently noted, with participants describing HCPs as encouraging and actively motivating them to achieve their goals as well as keeping them accountable.

“I did feel a little bit embarrassed because I was doing, like, planks on the floor and stuff like that. And when she started doing it with me, I felt like … I’m not embarrassed anymore” (P1).

“They always do, even if I’m not going to see the dietitian and I’m going to see the lady that I see for exercise, the dietitian will always bring me in for five minutes….just check in how I’m doing and stuff…. I always feel like the doctors, they always make sure that you’re doing good, you’re staying on track” (P5).

Participants felt that their care plans were tailored to their individual needs, which helped them stay on track with their health and fitness goals.

“They motivated me a lot and made plans that were suitable for me which was good” (P4).

“I mean, the plans made by the dietitian and everything has helped me so much” (P5).

“The exercises and advice was something that I was able to do …I think it’s generally good” (P3).

One participant described how switching to the current service had been the catalyst for improvement.

“Trying a new service back then was so much more better than keep on going to the same person that I was going to before. But coming to your service, it has helped me so much through the past, I think, three months I’ve been there. I’ve had so much help, so much improvements” (P5).

### Theme 3: impact

This theme is divided into two subthemes, focusing on participants’ clinical outcomes and overall wellbeing as a result of engaging with the service.

#### Subtheme 1: clinical outcomes

This subtheme was identified to reflect participants’ clinical outcomes from engaging with the service, including weight loss and improved biochemical markers. Participants reported a range of positive clinical outcomes due to their engagement. Several participants noted that the healthcare service had helped them in their weight loss journey.

“It has helped me lower my BMI” (P12).

“I’ve found it very helpful to lose weight and become healthier as a person” (P3).

“It’s made me lose a bit of weight… gave me more confidence” (P8).

Many participants expressed feeling like a “new person” and experiencing significant improvements in their overall health. They mentioned having more energy, higher stamina, and an overall sense of better health.

“I feel like a new person” (P1).

“My blood level is normal now; my eating habits are getting a little bit better“ (P2).

”Feel healthier and have higher stamina and more energy. Moving more and eating better” (P22).

For some, engaging with the service also encouraged them to become more physically active and improve their physical fitness.

“When I go up the stairs I don’t get tired… like my heart rate doesn’t go to like a hundred” (P1).

Additionally, they described having a better understanding of their diet. They appreciated the guidance on healthier eating habits and portion control.

“She pretty much opened my eyes to how much calories are in foods” (P11).

“It’s helped me, to have an understanding of the diet, give me different ways or like different proteins and all that stuff”(P3).

The education they received about nutrition and exercise was particularly valuable as it helped them stay on track and motivated them to make lasting positive lifestyle changes.

“It helped me keep on track and what I needed to do. Yeah, it helped me quite a bit. A lot more healthier“(P18).

##### Subtheme 2: emotional and social wellbeing

This subtheme was identified to reflect the participants’ emotional, psychological and social wellbeing. Some participants described how the positive changes in their health and appearance increased their confidence. They felt better about themselves and their abilities. Many participants received compliments and positive feedback from friends and family about their progress and achievements, which increased their self-esteem.

“You guys actually helped me and got me to the good shape. Gave me more confidence” (P19).

“I get all these compliments from people. Like you’ve done so well. It just feels great, you know” (P1).

“It’s made me lose a bit of weight… gave me more confidence” (P 8).

### Theme 4. Recommendations

This theme delves into participants’ feedback and suggestions for improving the service, within two subthemes: service feedback and improvement suggestions.

#### Subtheme 1. Service feedback

This subtheme was identified to reflect participants’ willingness to recommend the service to others. Even those who did not fully achieve their desired clinical outcomes expressed a strong likelihood of recommending the service. They related this to their positive experiences and the support they received.

“Definitely yes. Because it worked on me, it might work on them?” (P8).

“I would definitely advise them your service out of any service that I’ve been with. Because your service helped me so much, so I would obviously want you guys to help someone else in need” (P5).

A few participants shared an instance of recommending the program to a friend, emphasising the positive impact and how the service helped them.

“I told him about this program that, like, worked for me and I told him how, like how I lost my weight and how they helped me.…like how it was cool, like they would go all about things that you eat and stuff like that” (P1).

“I’ve been recommending you. Because I’ve lost so much weight. It just improved everything” (P4).

#### Subtheme 2. Improvement suggestions

This subtheme was identified to reflect participants’ suggestions for service, across various aspects.

Overall, most participants seemed satisfied with the current service delivery. They did not perceive a need for significant improvement. One participant said:

“Well, I think that…at the moment, the service is perfectly fine…. It didn’t really …it doesn’t really need to improve” (P2).

Others provided valuable feedback for service enhancement with some participants recommending improving the initial engagement by having staff introduce themselves before the first appointment and making the experience more welcoming.

“I think you spoke to my mum…It would be helpful if you introduced yourself to me before you came in” (P20).

They expressed flexibility in scheduling appointments and recommended offering after-school or weekend appointments for greater accessibility especially for those with busy schedules or specific needs.

“After school appointments or appointments on the weekend… the weekend appointments, just in case, like, kids have, like, a learning disability and they, like, really need help and then they’re missing like that gap of school, because it’s the only time you guys have… That way my mum doesn’t have to take my siblings with her” (P22).

There was a suggestion for home visits to be introduced allowing staff to better understand their home environment and personal challenges.

“Maybe home visits yeah, I guess it would be kind of a good way to get the staff members to understand the environment of the clients, where they live, how they… like you know” (P10) “Home-visiting…. because people might not have the same equipment that they normally use for the home” (P22).

Currently, the GHK service does not provide access to gym facilities, with some participants suggested would greatly enhance their ability to engage in exercise sessions. They expressed that having access to gym equipment typically found in commercial gyms would allow for more effective and structured exercises. One participant highlighted the challenges of not having equipment or guidance at their disposal.

“Definitely beneficial, cause for him to have gym equipment that commercial gyms have, pretty much what it does is, I tried to go to a gym… And she just told me, told me what machines to hop on, you know what machines I can go on. Nobody was there for me to show me what I’m meant to do on the machines… I had to self-learn that” (P10).

“A gym or like an exercise area that’s dedicated for exercise” (P22).

Towards the end of the program, participants typically receive less frequent appointments, which some found challenging. One participant said they would prefer more frequent follow-ups during the service’s later stages.

“I was seen less towards the end… more challenging: cause I didn’t get updates” (P8).

“Back then I was having like a session every month or like two months. So, there wasn’t much like you know, accountability for me to do anything, there wasn’t something to look forward to. So, I would have my session, take down notes, and do what I was meant to do, like for a week or two and then the third week would come and I’d be like why am I doing this again?” (P10).

A common suggestion from participants was the need for ongoing support beyond the usual 24-month program. Currently once the GHK program is completed there is no further contact with participants. However, several participants indicated that they would return for additional help if they experienced weight gain or difficulty maintaining their progress.

“Oh yeah, I guess so because I need to be able to maintain my weight. So I already know, like, strategies to, like, lose weight, but if that’s not working then I think being able to contact you guys again would be helpful” (P4).

Some suggested having a direct line to schedule appointments as required which would offer more flexibility.

“It would be more of, like, we have a number to be able to access them, to book an appointment. To be able to have access and to see what is needed, how to solve this issue that I’m having” (P3).

Interestingly, contrary to the strong preference for individual sessions throughout the service, a few participants were open to group sessions if further support was needed, or once they had made progress and felt more comfortable.

“Probably, if it was further on, as a group” (P18).

“Groups, with kids the same age as me” (P8).

“Groups…you can help more people at once” (P12).

Suggestions for the frequency of check-ups ranged from monthly to annually, depending on the individual needs and circumstances. Some participants saw the value of occasional check-ups after completing the service.

“Every few months” (P14).

“I guess every six months, something like that” (P20).

“The perfect period would maybe be …honestly maybe like just one year” (P1).

“Yeah to make sure I’m maintaining… Like maybe like, every year or something” (P21).

These themes illustrate the complex nature of clients’ experiences with the GHK service. They highlight the importance of clear communication, personalised support, and client-centered care, which is flexible and responsive in enhancing engagement and promoting positive outcomes in these young people.

## Discussion

This study identified valuable insights into the experiences of adolescents participating in this multidisciplinary paediatric weight management service in SWSLHD. Overall, the participants expressed a diverse range of emotions and expectations, with a preference for face-to-face, individual consultations with HCPs. They generally had positive experiences with the GHK service and were willing to recommend the service to others. Suggestions for service improvement included longer-term support, greater appointment flexibility, and access to gym equipment.

Participants in this study, like those in other paediatric weight management services, initially experienced nervousness, apprehension, stress, and anxiety [14, 20, 21]. Judgement related to weight and appearance is a prominent concern, reflecting the stigma associated with weight management services [22]. There is evidence that some children and their families avoid seeking weight management services altogether due to concerns about judgment and stigma [15]. Despite these concerns, the participants continued with the program, contrasting findings from Cox et al. [21] which linked initial apprehension as a barrier to accessing services. A positive emotional shift occurred during the first appointment when participants received more information about the service, with nonjudgemental and approachable clinicians playing a significant role in reducing anxiety. To support engagement, it is crucial to provide age-appropriate and clear information to both adolescents and their families before the initial appointment. Further research is needed to determine whether changing initial contact to be more adolescent focused improves client experience and outcomes.

This study identified that nearly all adolescents preferred one-on-one consultations over group setting, a preference supported by other research on service delivery models [23]. Participants cited comfort as the key factor, with experiences of bullying, social anxiety and emotional challenges as barriers to group intervention. While group interventions are more cost-effective [24, 25], they may not address individual needs, potentially impacting clinical outcomes. Compared with group-only approaches, mixed-format interventions which combine both group and individual sessions, achieve better outcomes by balancing individualised care with resource constraints [26]. However, there is limited consensus on best practices in service delivery for managing paediatric obesity [27]. Tailoring delivery methods is critical for addressing the complexities of paediatric obesity as well as decreasing attrition, with further research needed to evaluate the effectiveness of different service delivery with this service-diverse and vulnerable population.

Person-centred care is recognised as a foundation for safe, high-quality healthcare [28, 29]. Participants highlighted principles of person-centred care, noting clinicians were approachable, respectful, and caring, all while maintaining a high level of professionalism, which helped build trust. They appreciated clear communication, individualised support, accountability and motivation to achieve their goals. This positive experience led all the participants to express a willingness to recommend the service to their peers.

The flexibility of service provision was important, with the majority of participants having a preference for face-to-face appointments, however, sometimes, it was viewed as disruptive due to competing priorities such as school attendance. Many participants requested more flexible appointment options, especially on weekends or after school, to minimise disruption. Similar studies have shown that limitations in clinic time and school/work commitment contribute to families discontinuing an obesity management service [30–32]. Participants found telehealth appointments beneficial, especially for those who struggled with in-person visits. Utilising telehealth interventions in conjunction with face-to-face sessions can reduce logistic barriers [33, 34]. A few of the participants suggested the option of a home visit, which would offer clinicians better insight into their social and physical environment, potentially facilitating a more tailored care plan. This aligns with findings from a qualitative study where 89% of families were interested in home visits [35]. While a systematic review show promising efficacy for home visits, further research is needed to assess their sustainability, effectiveness and feasibility, especially considering resource constraints [36].

The American Psychological Association (APA) recommended at least 26 contact hours of intensive behavioural intervention for effective weight loss in children over a 12-month period [27]. Long-term monitoring and follow-up increase the chance of sustained lifestyle changes and weight loss for 2 to 5 years [37–39]. A systematic review found that participants in paediatric weight management services prefer longer-term support [14], with only 2 out of the 26 studies having interventions lasting 24 months or longer. Despite the GHK service being a 24-month intensive program, participants still reported they would benefit from longer-term support in the form of periodic monitoring, mainly to prevent and manage weight regain. They prefer ongoing support via face-to-face appointments and/or telehealth, and a few participants expressed interest in a group format. Balancing contact frequency with sustainability and resource constraints is essential with the potential of using group settings and telehealth for long-term follow-up given their effectiveness in maintaining weight loss [40–43].

Participants indicated that having more resources, such as access to gym and gym equipment at clinic locations would be beneficial, as it would help facilitate their exercise sessions. This finding is supported by a qualitative systematic review stating that active engagement with hands on activities are of large importance to participants when attending an obesity intervention [14]. Providing access to a commercial gym setting within the program might also be valuable in helping children transition to self-care, by offering them a more structured and supportive environment to develop long-term physical habits.

The findings of this qualitative study highlight several important considerations for improving the experience of adolescents accessing weight management services. They reinforce the importance of a multidisciplinary and person-centred approach to care that addresses the individual needs of each participant. The study also emphasises the need to reduce participants’ initial anxiety, which can act as a barrier to engagement, by providing pre-service resources that introduce the service and clearly outline expectations in a way that is tailored to adolescents. Having clinicians who are trained in paediatric obesity management may play a role in providing a non-judgemental environment for adolescents, reducing the risk of stigma associated with weight management services. This may help with addressing the initial anxiety of the adolescents, which may improve their overall experience and engagement of the service. Although group-based interventions may offer greater efficiency, they may not meet the specific needs of the target client group and should therefore be considered in the context of available resources. Furthermore, the study highlights that service delivery can be improved to enhance engagement. This includes increasing the availability of after school appointments, incorporating telehealth, exploring the feasibility of home visits, and offering long term support.

The strength of this study lies in its commitment to diversity, featuring participants from various backgrounds, including adolescents of CALD and Aboriginal status. This representation mirrors the demographics of the SWSLHD population and GHK clients, who are frequently overlooked in research [16]. Our research examined a comprehensive 24 month paediatric obesity management service, collecting valuable insights from participants throughout different stages. Currently, there is a notable scarcity of qualitative studies focused on adolescents engaged in paediatric weight management service of 24 months or longer [44, 45]. By ensuring that interviews were conducted by experienced clinicians who were not involved in the participants’ treatment, we effectively minimised bias and facilitated open, honest communication.

A limitation of this study is the exclusion of participants who withdrew from or disengaged from the program. Addressing this gap by exploring the factors behind attrition could yield essential insights for improving our service. While most interviews were conducted by non-treating clinicians to minimise bias, there is still a risk that interpretations of questions and the elicitation of responses may be influenced in subtle ways. Furthermore, participants’ awareness of the interviewer’s connection to the service, along with the presence of parents during some interviews, could have hindered their willingness to openly share their perspectives and discuss sensitive topics. We also acknowledge the increased risk of bias in one interview that was conducted by the treating clinician. More extensive and in-depth studies should be conducted with participants from diverse backgrounds to ensure findings of this study are relevant and applicable to a wider population, and for addressing health disparities, potentially leading to more effective and equitable outcomes.

## Conclusion

This study identified the emotional complexities participants face when engaging in weight management services, such as nervousness, stress, and concerns about judgment. However, these challenges can be mitigated by clear communication, empathetic care, and supportive environments. By offering long-term support, flexible follow-up, and multiple modalities for care delivery, the experience, outcomes and engagement of adolescents living with obesity can be greatly enhanced. Overall, the GHK paediatric weight management service was positively received, and feedback gained from understanding participants’ experiences offers meaningful ways to improve the existing services.

## Supplementary Information

Supplementary Material 1. Appendix A: Interview guide: Children and young people (12 to 17 years of age).Semi-structured interview guide used to explore the experiences and perspectives of children and young people participating in the study. The guide includes open-ended questions and suggested prompts used during interviews

## Data Availability

No datasets were generated or analysed during the current study.

## References

[1] Australian Bureau of Statistics. Waist circumference and BMI 2022 [Available from: https://www.abs.gov.au/statistics/health/health-conditions-and-risks/waist-circumference-and-bmi/latest-release

[2] Mehdi SP, Pasricha J, Biggs BA. Hyperlipidaemia and weight amongst Afghani refugees attending a general practice clinic in regional Australia. J Immigr Minor Health. 2023;25(3):589–95. 36745279 10.1007/s10903-022-01446-1 PMC10212845

[3] Broyles ST, Denstel KD, Church TS, Chaput JP, Fogelholm M, Hu G, et al. The epidemiological transition and the global childhood obesity epidemic. Int J Obes Suppl. 2015;5(Suppl 2):S3–8. 27152182 10.1038/ijosup.2015.12 PMC4850613

[4] Hardy LL, Jin K, Mihrshahi S, Ding D. Trends in overweight, obesity, and waist-to-height ratio among Australian children from linguistically diverse backgrounds, 1997 to 2015. Int J Obes (Lond). 2019;43(1):116–24. 29980760 10.1038/s41366-018-0139-5 PMC6331387

[5] Australian Bureau of Statistics. 2021 Sydney- South West, Census All persons QuickStats Canberra: Australian Bureau of Statistics; 2021 [Available from: https://www.abs.gov.au/census/find-census-data/quickstats/2021/127

[6] Owen K, Corbett C, Thomas M. Growing healthy kids in South West sydney: results of the 2018 baseline population health Survey. Final Report. The university of Sydney. Sydney: Prevention Research Collaboration, Sydney School of Public Health,; 2021.

[7] Kansra AR, Lakkunarajah S, Jay MS. Childhood and adolescent obesity: A review. Front Pediatr. 2020;8:581461. 33511092 10.3389/fped.2020.581461 PMC7835259

[8] Moradi M, Mozaffari H, Askari M, Azadbakht L. Association between overweight/obesity with depression, anxiety, low self-esteem, and body dissatisfaction in children and adolescents: a systematic review and meta-analysis of observational studies. Crit Rev Food Sci Nutr. 2021;62(2):555–70. 10.1080/10408398.2020.1823813 32981330

[9] Cheng S, Kaminga AC, Liu Q, Wu F, Wang Z, Wang X, et al. Association between weight status and bullying experiences among children and adolescents in schools: an updated meta-analysis. Child Abuse Negl. 2022;134:105833. 36219907 10.1016/j.chiabu.2022.105833

[10] Southcombe F, Vivekanandarajah S, Krstic S, Lin F, Chay P, Williams M, et al. More than just body mass index: using the Edmonton obesity staging system for pediatrics to define obesity severity in a multi-ethnic Australian pediatric clinical cohort. Obes Sci Pract. 2023;9(3):285–95. 37287524 10.1002/osp4.648 PMC10242255

[11] Simmonds M, Llewellyn A, Owen CG, Woolacott N. Predicting adult obesity from childhood obesity: a systematic review and meta-analysis. Obes Rev. 2016;17(2):95–107. 26696565 10.1111/obr.12334

[12] Southcombe F, Krstic S, Lin F, Vivekanandarajah S, Williams M, Chay P, Cheng H, Rahman Khan J, Eapen V, Dennis S, Denney-Wilson E, Lingam R. Growing healthy kids: results of a quasi-experimental evaluation of a pediatric weight management service. Obesity Science & Practice, under review 2024.

[13] Ross MM, Kolbash S, Cohen GM, Skelton JA. Multidisciplinary treatment of pediatric obesity: nutrition evaluation and management. Nutr Clin Pract. 2010;25(4):327–34. 20702836 10.1177/0884533610373771 PMC3977477

[14] Jones HM, Al-Khudairy L, Melendez-Torres GJ, Oyebode O. Viewpoints of adolescents with overweight and obesity attending lifestyle obesity treatment interventions: a qualitative systematic review. Obes Rev. 2019;20(1):156–69. 30375160 10.1111/obr.12771

[15] Hampl SE, Hassink SG, Skinner AC, Armstrong SC, Barlow SE, Bolling CF, et al. Clinical Practice Guideline for the Evaluation and Treatment of Children and Adolescents With Obesity. Pediatrics. 2023;151(2). 10.1542/peds.2022-060640 36622115

[16] Kelly R, Hatzikiriakidis K, Kuswara K. Inequities in obesity: Indigenous, culturally and linguistically diverse, and disability perspectives. Public Health Res Pract. 2022;32(3):e3232225. 10.17061/phrp3232225. 10.17061/phrp3232225 36220561

[17] Doyle L, McCabe C, Keogh B, Brady A, McCann M. An overview of the qualitative descriptive design within nursing research. J Res Nurs. 2019;25(5):443–55. 34394658 10.1177/1744987119880234 PMC7932381

[18] Pham TTL, Berecki-Gisolf J, Clapperton A, O’Brien KS, Liu S, Gibson K. Definitions of Culturally and Linguistically Diverse (CALD): A Literature Review of Epidemiological Research in Australia. Int J Environ Res Public Health. 2021;18(2). 10.3390/ijerph18020737 PMC7830035 33467144

[19] Nowell LS, Norris JM, White DE, Moules NJ. Thematic analysis: striving to Meet the trustworthiness criteria. Int J Qualitative Methods. 2017;16(1):1609406917733847.

[20] Banks J, Cramer H, Sharp DJ, Shield JP, Turner KM. Identifying families’ reasons for engaging or not engaging with childhood obesity services: A qualitative study. J Child Health Care. 2014;18(2):101–10. 23728931 10.1177/1367493512473854

[21] Cox JS, Searle AJ, Hinton EC, Giri D, Shield JPH. Perceptions of non-successful families attending a weight-management clinic. Arch Dis Child. 2021;106(4):377–82. 33139347 10.1136/archdischild-2020-319558

[22] Puhl RM. Weight stigma and barriers to effective obesity care. Gastroenterol Clin. 2023;52(2):417–28. 10.1016/j.gtc.2023.02.002 37197883

[23] Smith KL, Straker LM, McManus A, Fenner AA. Barriers and enablers for participation in healthy lifestyle programs by adolescents who are overweight: a qualitative study of the opinions of adolescents, their parents and community stakeholders. BMC Pediatr. 2014;14(1):53. 24552207 10.1186/1471-2431-14-53 PMC3942615

[24] Barlow SE, Committee atE. Expert committee recommendations regarding the Prevention, Assessment, and treatment of child and adolescent overweight and obesity: summary report. Pediatrics. 2007;120(Supplement4):S164–92. 18055651 10.1542/peds.2007-2329C

[25] Goldfield GS, Epstein LH, Kilanowski CK, Paluch RA, Kogut-Bossler B. Cost-effectiveness of group and mixed family-based treatment for childhood obesity. Int J Obes Relat Metab Disord. 2001;25(12):1843–9. 11781766 10.1038/sj.ijo.0801838

[26] Hayes JF, Altman M, Coppock JH, Wilfley DE, Goldschmidt AB. Recent updates on the efficacy of group based treatments for pediatric obesity. Curr Cardiovasc Risk Rep. 2015;9(4):16. 25866596 10.1007/s12170-015-0443-8 PMC4389784

[27] Tully L, Arthurs N, Wyse C, Browne S, Case L, McCrea L, et al. Guidelines for treating child and adolescent obesity: A systematic review. Front Nutr. 2022;9:902865. 36313105 10.3389/fnut.2022.902865 PMC9597370

[28] Australian Commision on Safety and Quality in Health Care. Person-centred care Australia2024 [Available from: https://www.safetyandquality.gov.au/our-work/partnering-consumers/person-centred-care

[29] The Health Foundation. Person-centred care made simple. London2016. Available from: https://www.health.org.uk/sites/default/files/PersonCentredCareMadeSimple.pdf

[30] Sallinen Gaffka BJ, Frank M, Hampl S, Santos M, Rhodes ET. Parents and pediatric weight management attrition: experiences and recommendations. Child Obes. 2013;9(5):409–17. 24028563 10.1089/chi.2013.0069

[31] Kwitowski M, Bean MK, Mazzeo SE. An exploration of factors influencing attrition from a pediatric weight management intervention. Obes Res Clin Pract. 2017;11(2):233–40. 27544283 10.1016/j.orcp.2016.08.002 PMC5315693

[32] Hampl S, Demeule M, Eneli I, Frank M, Hawkins MJ, Kirk S, et al. Parent perspectives on attrition from tertiary care pediatric weight management programs. Clin Pediatr (Phila). 2013;52(6):513–9. 23539682 10.1177/0009922813482515

[33] Kouvari M, Karipidou M, Tsiampalis T, Mamalaki E, Poulimeneas D, Bathrellou E, et al. Digital health interventions for weight management in children and adolescents: systematic review and Meta-analysis. Journal of Medical Internet Research; 2022. 10.2196/30675 PMC8887634 35156934

[34] Qiu L-T, Sun G-X, Li L, Zhang J-D, Wang D, Fan B-Y. Effectiveness of multiple eHealth-delivered lifestyle strategies for preventing or intervening overweight/obesity among children and adolescents: A systematic review and meta-analysis. Front Endocrinol. 2022;13:999702. 10.3389/fendo.2022.999702 PMC9491112 36157474

[35] Gehring ND, Ball GDC, Perez A, Holt NL, Neuman D, Spence N, et al. Families’ perceived benefits of home visits for managing paediatric obesity outweigh the potential costs and barriers. Acta Paediatr. 2018;107(2):315–21. 28960483 10.1111/apa.14101

[36] Appelhans BM, Moss OA, Cerwinske LA. Systematic review of paediatric weight management interventions delivered in the home setting. Obes Rev. 2016;17(10):977–88. 27231126 10.1111/obr.12427

[37] National Health and Medical Research Council. Clinical practice guidelines for the management of overweight and obesity in adults, adolescents and children in Australia. VIC, Australia: NHMRC; 2013.

[38] Ek A, Brissman M, Nordin K, Eli K, Nowicka P. A long-term follow-up of treatment for young children with obesity: a randomized controlled trial. Int J Obes (Lond). 2023;47(11):1152–60. 37723272 10.1038/s41366-023-01373-7 PMC10599998

[39] Epstein LH, Valoski A, Wing RR, McCurley J. Ten-year outcomes of behavioral family-based treatment for childhood obesity. Health Psychol. 1994;13(5):373. 7805631 10.1037//0278-6133.13.5.373

[40] Cussler EC, Teixeira PJ, Going SB, Houtkooper LB, Metcalfe LL, Blew RM, et al. Maintenance of weight loss in overweight middle-aged women through the internet. Obes (Silver Spring). 2008;16(5):1052–60. 10.1038/oby.2008.19 18309301

[41] Flodgren G, Deane K, Dickinson HO, Kirk S, Alberti H, Beyer FR, et al. Interventions to change the behaviour of health professionals and the organisation of care to promote weight reduction in overweight and obese people. Cochrane Database Syst Rev. 2010;(3):Cd000984. 10.1002/14651858.CD000984.pub2 PMC4235843 20238311

[42] Neve M, Morgan PJ, Jones PR, Collins CE. Effectiveness of web-based interventions in achieving weight loss and weight loss maintenance in overweight and obese adults: a systematic review with meta-analysis. Obes Rev. 2010;11(4):306–21. 10.1111/j.1467-789X.2009.00646.x 19754633

[43] Svetkey LP, Stevens VJ, Brantley PJ, Appel LJ, Hollis JF, Loria CM, et al. Comparison of strategies for sustaining weight loss: the weight loss maintenance randomized controlled trial. JAMA. 2008;299(10):1139–48. 18334689 10.1001/jama.299.10.1139

[44] Campbell-Voytal KD, Hartlieb KB, Cunningham PB, Jacques-Tiura AJ, Ellis DA, Jen KC, et al. African American Adolescent-Caregiver relationships in a weight loss trial. J Child Fam Stud. 2018;27(3):835–42. 29610562 10.1007/s10826-017-0920-4 PMC5877412

[45] Hammar SL, Campbell V, Woolley J. Treating adolescent obesity. Long-range evaluation of previous therapy. Clin Pediatr (Phila). 1971;10(1):46–52. 5545911 10.1177/000992287101000117

