# Supplementary material for: Youth perspectives: experiences with a multidisciplinary weight management service, a qualitative study
Source: BMC Pediatr. 2025 Nov 18;25:939. doi: 10.1186/s12887-025-06239-7 (PMC12625158; doi:10.1186/s12887-025-06239-7)
Supplement: Supplementary file 1 — Supplementary Material 1. Appendix A: Interview guide: Children and young people (12 to 17 years of age).Semi-structured interview guide used to explore the experiences and perspectives of children and young people participating in the study. The guide includes open-ended questions and suggested prompts used during interviews [file 12887_2025_6239_MOESM1_ESM.docx]

**APPENDIX A**

**Interview guide: Children and young people (12** **–17 years old)**

We are interested in your views on how you will engage with the Growing Healthy Kids service, how you think it can help you and what we could improve on.

1. **How did you feel about coming to the Growing Healthy Kids service?**

Prompts

- Did you have any feelings of anticipation? Stress? Excitement? Why?
- Can you tell me a bit more about that?
- Is there anything that we could have done as a service to make you feel better/ more comfortable?

1. **Did you think you needed to see the service?**

Prompts

- Why or why not?
- Did other people think you need the service? Who are these people? How confident were these people that the service will work?
- Did other people think you need the service? Who are these people?
- How confident were these people that the service will work?

1. **What did you like most about the GHK service? What was most helpful? What was least helpful?**

Prompts

- What about individual sessions? Or group sessions?
- Did you get enough time and meetings with the team? Was it too many or not enough?
- How was the location? Or the time of the day?
- Did you get help finding other services (e.g. exercise centres, referrals to other health care)?
- Did you find the Health Care Staff helpful? Approachable? Respectful? Did you feel listened too?
- Have you tried other weight services (Go4Fun, GP, Dietitian)? What was different about GHK service?

1. **What would make the Growing Health Kids service better?**

Prompts

- Could we improve the groups? What about individual sessions?
- What about when you finished the service, would you find it helpful to still have access to support? Prompt: Individual or group? Would you like us to call you for a check-in or allowing clients to call us whenever as required.
- Could it be delivered differently? Home visits, What about video calls or other social media?
- How did you find the facilities? Exercise equipment, clinic rooms etc.

1. **Has being a part of the Growing Healthy Kids service helped?**

Prompts

- If so, how do you think the service has helped?
- And how do you feel about your health now since being with the service? Do you feel like your health has improved after joining the GHK service?
- Have you had any lifestyle changes because of being part of the service?
- Would you recommend our service to your friends? Why or why not?

1. **Is there anything else that you would like to add?**
